# Transcriptomic Profiling at the Maternal-to-Zygotic Transition in Leech, *Helobdella austinensis*

**DOI:** 10.3390/genes15030283

**Published:** 2024-02-24

**Authors:** Samuel Hsaio, Naim Saglam, David Morrow, Daniel H. Shain

**Affiliations:** 1 Center for Computational and Integrative Biology, Rutgers The State University of New Jersey, Joint Health Sciences Center, 201 South Broadway, Camden, NJ 08103, USA; 2 Department of Aquaculture and Fish Diseases, Fisheries Faculty, Firat University, 23200 Elazig, Türkiye; 3 Biology Department, Rutgers The State University of New Jersey, Joint Health Sciences Center, 201 South Broadway, Camden, NJ 08103, USA

**Keywords:** glossiphoniid, development, maternal RNA, differential expression, fertilization

## Abstract

The glossiphoniid leech, *Helobdella austinensis*, is an experimentally tractable member of the superphylum, Lophotrochozoa. Its large embryonic cells, stereotyped asymmetric cell divisions and ex vivo development capabilities makes it a favorable model for studying the molecular and cellular events of a representative spiralian. In this study, we focused on a narrow developmental time window of ~6–8 h, comprising stages just prior to and immediately following zygote deposition. Employing RNA-Seq methodology, we identified differentially expressed transcripts at this fundamental ontogenic boundary, known as the maternal-to-zygotic transition (MZT). Gene expression changes were characterized by the massive degradation of maternal RNAs (~45%) coupled with the rapid transcription of ~5000 zygotic genes (~20% of the genome) in the first mitotic cell cycle. The latter transcripts encoded a mixture of cell maintenance and regulatory proteins that predictably influence downstream developmental events.

## 1. Introduction

Bilaterians fall into three major taxonomic clades, the Deuterostomia (e.g., *Danio rerio*, *Mus musculus*, *Xenopus laevis*), Ecdysozoa (e.g., *Drosophila melanogaster*, *Caenorhabditis elegans*), and Lophotrochozoa (e.g., annelids, molluscs) [1,2]. Commonly studied metazoans fall almost exclusively into the former two “superphyla” (i.e., Deuterostomia, Ecdysozoa). Thus, the incorporation of lophotrochozoan models into scientific exploration provides an important perspective on topics relating to the evolution of genes and genomes, developmental mechanisms, and biological novelties.

Clitellate annelids (i.e., oligochaetes, including leeches) offer a number of unique features as experimental lophotrochozoan model systems. In particular, species of the dorsoventrally flattened glossiphoniid leech genus, *Helobdella*, lay unusually large (~400 μm diameter), lethicotrophic eggs that develop ex vivo and thus are amenable to experimental manipulation. Moreover, the early cell divisions are rapid, asymmetric, and stereotypical, such that individual cell types can be identified and isolated to homogeneity [3,4]. Leech development is characterized by early spiral cleavages leading to five bilateral pairs of lineage-specific stem cells, which thereafter give rise to the segmental mesoderm and ectoderm [5]. Coupled with their relatively small genome size and the availability of annotated reference genomes, *Helobdella* spp. are thus well suited for investigating the genetic factors that guide early cell divisions and later developmental events.

The leech, *H. austinensis*, is a representative *Helobdella* species that is readily propagated in a laboratory setting [6,7]. Its generation time is ~10 weeks at 23 °C, and clutch sizes range from ~30 to 80 embryos, which facilitates the acquisition of sufficient starting material for standard molecular and cellular analyses. With the availability of powerful, high-throughput sequencing technologies (e.g., RNA-Seq), we aimed to take advantage of accessible *H. austinensis* cells immediately prior to and following zygote deposition (i.e., egg laying), in an effort to identify changes in gene expression at this critical ontogenic boundary (i.e., the maternal-to-zygotic transition; MZT). Specifically, oocytes accumulate along the ventral midline in oviducts and are fertilized internally before passing through the female gonopore. The first cell division occurs within ~4 h after zygote deposition and is asymmetric, indicating that polarity is established early, and possibly exists in the unfertilized egg [8,9,10].

By harvesting eggs immediately prior to and just following zygote deposition—cell types separated by only a few hours of development—we were able to monitor two types of differentially expressed RNA transcripts, maternal RNAs and those first transcribed in the zygote. Taken as a whole, this RNA profile (maternal + zygotic) comprises the genetic framework and starting point for directing embryogenesis in *H. austinensis*, a genetically tractable and experimentally amenable representative of the superphylum, Lophotrochozoa.

## 2. Materials and Methods

### 2.1. Specimens and Cell Acquisition

**Figure 1:**
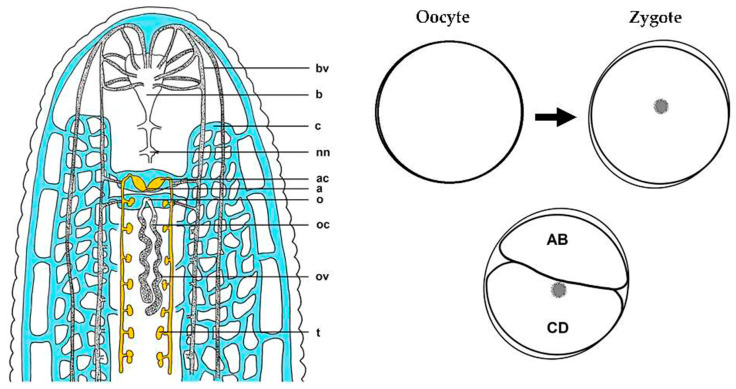
Schematic of *H. austinensis* showing reproductive structures and early developmental stages. Like all leech species, *H. austinensis* is a hermaphrodite, containing both ovaries and testes. Semen produced in the testes is transmitted to the atrial cornua via the seminal ducts and covered with a sheath to form the spermatophore, which travels to the male genital atrium and enters the coelomic cavity of another leech following traumatic insemination. Spermatozoa enter the ovary through the walls of the female genital organs and fertilize oocytes. Unfertilized oocytes in oviducts represent Stage 0 (but note that internally fertilized zygotes remain arrested in meiosis for several hours prior to deposition, as reviewed in [9]); fertilized eggs represent the zygote and the presumptive onset of zygotic transcription immediately following deposition (Stage 1). The first mitotic cell division is asymmetric, yielding a smaller AB and larger CD blastomere, respectively. The teloplasm (yolk-free cytoplasm shown as a gray spot) partitions mostly to the CD cell. Outer circles surrounding cells identify the protective vitelline envelopes. Images not drawn to scale. a—atrium; ac—atrial crop; b—brain; bv—blood vessel; c—coelom; nn—nerve node (ganglion); o—oviduct; oc—oocyte; ov—ovarium; t—testis.

A colony of glossiphoniid leeches, *H. austinensis*, was maintained in glass bowls at 23 °C and fed a diet of freshwater snails and insect larvae, as described [6,7]. Stage 0 (prior to zygote deposition) and Stage 1 (following zygote deposition) cells were harvested from adult specimens prior to and shortly after egg laying, respectively (Figure 1). Oocytes were dissected from gravid leeches with a scalpel and fine pins within ~4 h of anticipated laying, as estimated through extensive observations of the process. Fertilized eggs were harvested within ~3 h after laying, prior to the asymmetric first cleavage (Figure 1). Clutches from both stages, ranging in size from 30 to 80 cells, were immediately transferred into guanidium (Solution 1; Total RNA Extraction Kit, ZYMO Research, Irvine, CA, USA), incubated for 1 h at room temperature to solubilize tissue and stabilize RNA, and then frozen at −80 °C for storage.

### 2.2. Transcriptome Construction and Analysis

Total RNA from pooled samples, containing at least 100 cells from each stage (i.e., Stages 0, 1), was extracted with a Total RNA Extraction Kit (ZYMO Research) according to manufacturer’s instructions. RNAs (three independent replicates at Stage 0, four independent replicates at Stage 1) were shipped on dry ice to Azenta (Plainfield, NJ, USA), which processed all samples for RNA-Seq, using the *Helobdella robusta* reference genome (NCBI taxis: 6412; http://genome.jgi-psf.org/Helro1/Helro1.home.html, accessed on 6 December 2023) for annotation. Transcripts were trimmed using Trimmomatic v.0.36 [11], then aligned using STAR v2.7.10b [12]. Transcriptomes included more than 2 million reads, with >12,000 unique transcripts aligning to the *H. robusta* genome. DeSeq2 [13] was conducted in RStudio v4.3.0 to determine most significant differentially expressed transcripts between cell types. Criterion for significance was set at two-fold difference between Stage 0 and Stage 1 transcript pools. Graphs were generated using Matplotlib v3.8.2 in Python3 v3.6.8. For plots with total transcript counts, average values from the various trials were recorded. Scatter plots were generated using average counts at Stage 0 and 1, with Log 10 applied to both axes.

### 2.3. Gene Searches

Transcripts with significant expression differences were translated and subjected to BLASTx searches against the NCBI protein database (version 2.15.0+). E-value thresholds were set at 1 × 10^−11^. Use of NCBI BLAST was conducted in Python3 using the Biopython API v1.82 with the NCBIWWW and NCBIXML module of the Bio.Blast package. Validity of this approach was verified by comparing BLAST results of upregulated genes by E-value, query coverage, and percentage of identity. Searches of known genes were performed with BLASTp v2.15.0 Protein sequences were acquired from model species (e.g., *D. melanogaster*) and blasted against the *H. robusta* reference genome, recording the top hit. The *H. austinensis* transcriptome was fully compared to *H. robusta*, thus allowing tracking of changes between stages and identification of homologues. To identify potential paralogues, the top 10 most closely related sequences of respective target genes were assessed to determine whether expression comparisons altered the results.

### 2.4. Classification Using KEGG

BLAST results were organized according to the Kyoto Encyclopedia of Genes and Genomes (KEGG), using the pathway database for *H. robusta*. Genes were classified according to the highest-level pathways in KEGG, namely, “Environmental Information Processing”, “Genetic Information Processing”, “Metabolism”, and “Cellular Processes”. “No match found” was recorded for genes with no records in KEGG. Access to KEGG was achieved using the “Keggtools” API in Python.

## 3. Results

**Figure 2:**
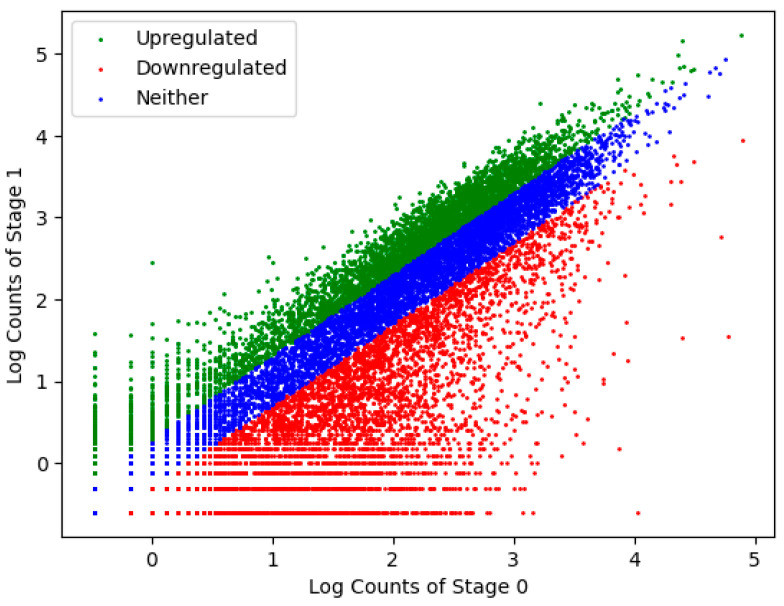
Scatter plot of unique transcripts according to Log−10 counts in Stage 0 vs. Stage 1, with a 2-fold cut-off threshold. Each dot identifies a single, unique transcript, color-coded according to the legend.

RNA transcripts from Stage 0 and Stage 1 were quantitated and compared computationally to determine immediate changes in the zygotic RNA profile following zygotic deposition. In total, ~18,954 unique assembled transcripts—representing ~80% of the full genome—were recorded from the combined analysis of the Stage 0 and Stage 1 *H. austinensis* transcriptomes. Among these, ~3322 were Stage 0-specific and 760 were Stage 1-specific, while a total of ~8295 were downregulated (i.e., degraded maternal mRNAs; ~45% of maternal pool) and ~4979 were upregulated (first zygotic transcripts; ~20% of total genome) based on a twofold threshold (Table 1, Figure 2). These values trended accordingly with 5- and 10-fold thresholds, respectively (Supplementary Table S1, Figure S1). Note that although more unique transcripts were downregulated, upregulated transcripts showed a greater magnitude of change: ~3-fold.

**Figure 3:**
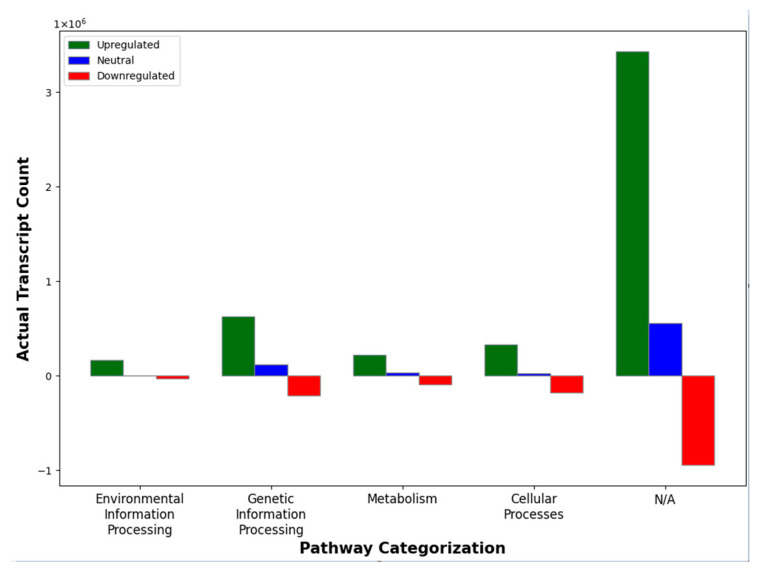
Differentially expressed transcripts classified according to KEGG pathways with a 2-fold threshold.

Among the ~18,954 uniquely identified *H. austinensis* transcripts, only 2889 matched an associated KEGG pathway. The categorized transcripts represented 110 different pathways, including endocytosis (158 transcripts), autophagy (114 transcripts), and protein processing in the endoplasmic reticula (112 transcripts). The total transcript counts were greater in upregulated vs. downregulated genes (Figure 3).

**Figure 4:**
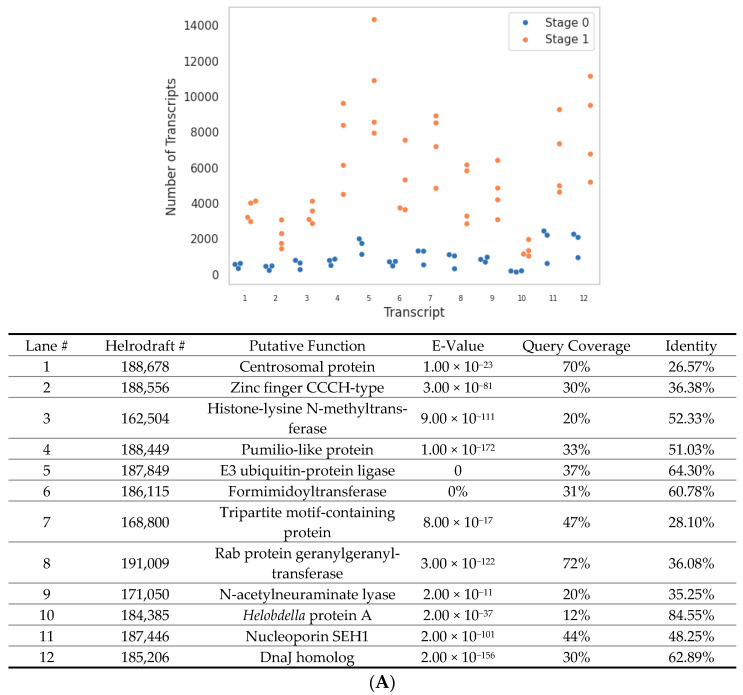

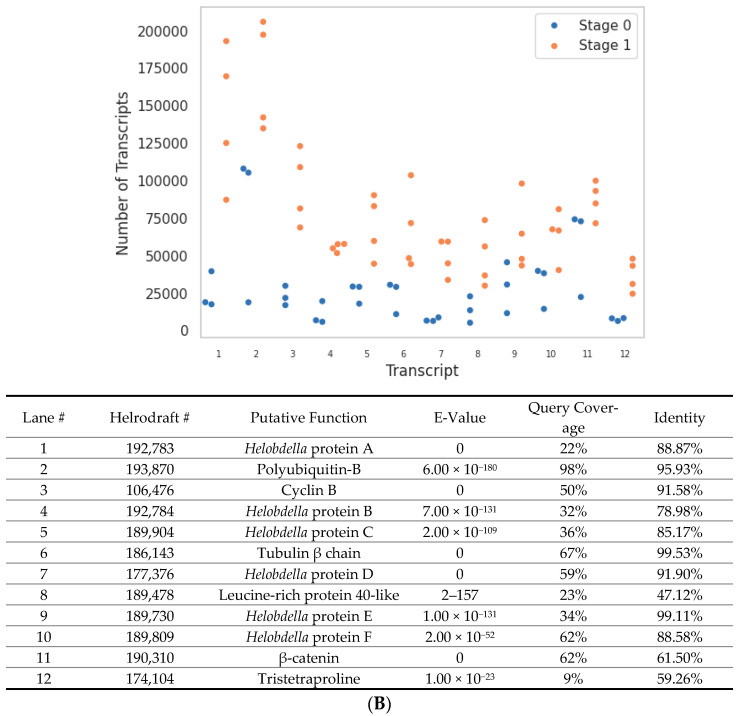

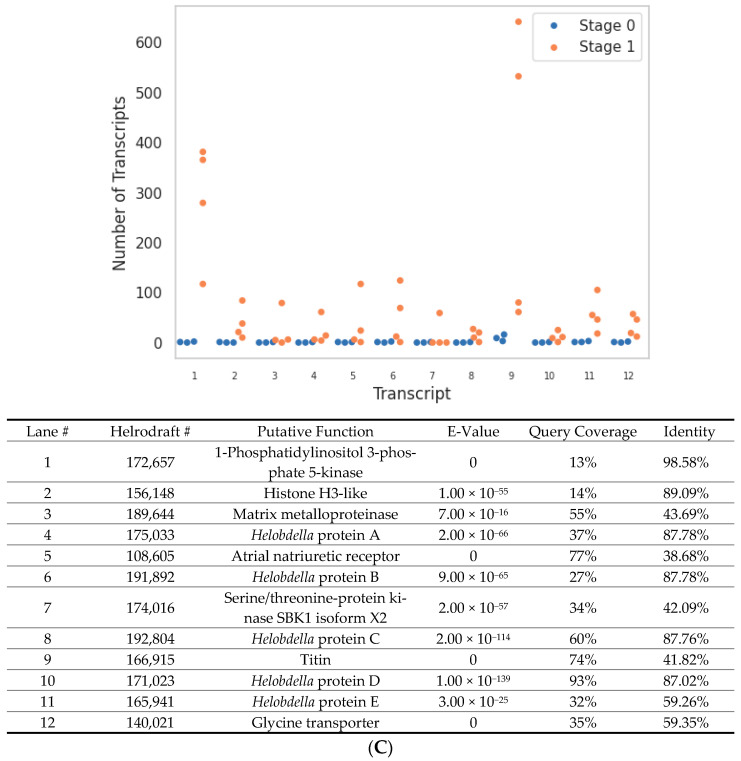
Differential expression of *H. austinensis* at the maternal-to-zygotic transition. (**A**) Twelve most upregulated zygotic genes according to DeSeq2 statistical analysis. Dots identify independent transcriptomes from Stage 0 and Stage 1, respectively. (**B**) Twelve most upregulated zygotic genes according to transcript count change. (**C**) Twelve most upregulated zygotic genes according to fold change.

To determine the upregulated candidate genes associated with early developmental events, we applied a rubric that employed three independent approaches: DeSeq2, the total count change between differentially expressed transcripts, and the fold change between Stage 0 and Stage 1 transcripts, respectively. Putative functions of candidate genes were predicted based on the top NCBI BLASTp hits and included a mixture of cell maintenance (e.g., centrosome, histone, tubulin, and cyclin-like) and regulatory (e.g., Pumilio, Tristetraproline, Nucleoporin, and zinc-finger-containing) genes (Figure 4). Comparable analyses were conducted on downregulated transcripts between Stages 0 and 1 (Supplementary Figure S2).

**Figure 5:**
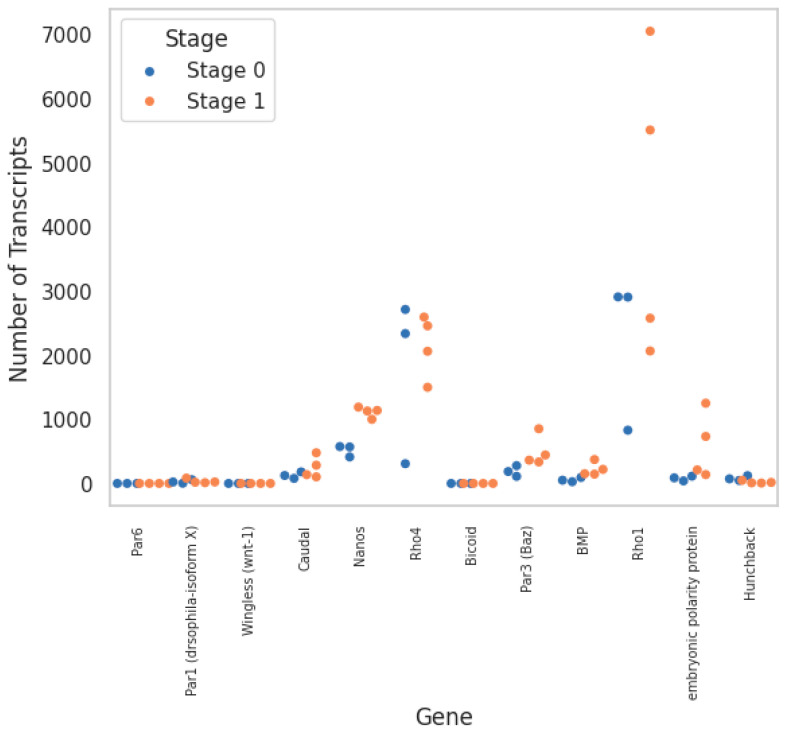
Transcript counts associated with each selected candidate gene based on BLASTp searches. Dots identify independent transcriptomes from Stage 0 and Stage 1, respectively.

**Figure 6:**
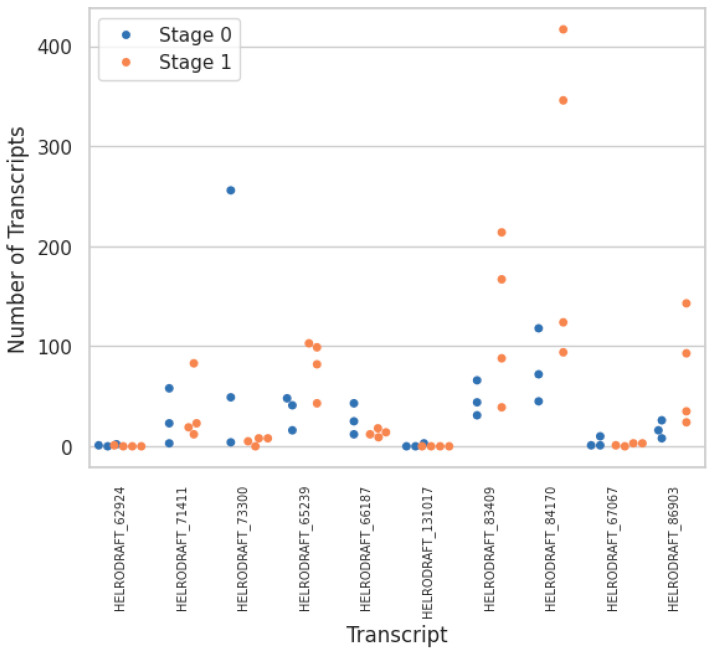
Transcript counts for BLASTp results of putative Par1 homologs. Dots identify independent transcriptomes from Stage 0 and Stage 1, respectively.

Applying a biased approach using known early developmental control genes yielded a selected list of 12 candidates that were compared against our transcriptomic data (Table 2; Figure 5). Among the selected candidates, about half were differentially expressed between Stages 0 and 1, while putative homologues for the remaining were not detected in our dataset. An expansion of the former group to include related sequences in *H. austinensis* showed that only Par1 had potential paralogues that were differentially expressed between cell types (Figure 6).

## 4. Discussion

The ability to monitor gene expression in its totality has led to both discovery and confusion, since datasets can be simultaneously revealing yet also overwhelming. Indeed, even subtle shifts in carbon source can lead to thousands of gene expression changes (e.g., [14]). It is therefore not too surprising that ~70% of RNA transcripts (~13,274 total identified transcripts) were differentially expressed in *H. austinensis* during the short maternal-to-zygotic (MZT) time window between Stage 0 and Stage 1 (separating zygotic deposition), totaling ~6–8 h. Nonetheless, it seems relevant that ~45% of the total maternal RNA pool was downregulated, including ~20% that was degraded in its entirety during this time interval. Similar values for maternal RNA clearance have been reported in flies (~60%), nematodes (~30%), and zebrafish at (25%) at the MZT [15,16,17]. This massive degradation of maternal RNAs appears to be wasteful in the context of oocyte resource expenditure but raises the possibility that oocytes stockpile ribonucleotides in the form of linear mRNAs, whose rapid hydrolysis yields a large pool of starting material for supporting zygotic transcription [18]. Considering that early embryonic cleavages in *Helobdella* occur rapidly (every few hours; [4]) and the demand for ribonucleotides is high (e.g., ~20% of genome activated upon fertilization but before the first cell division), the sequestration of maternal RNAs may be energetically strategic in terms of facilitating embryogenesis. Note that the onset of *H. austinensis* zygotic transcription within the first mitotic cell cycle is atypical in comparison with other metazoans (e.g., flies, frogs nematodes, zebrafish), in which the process typically begins between cell cycles 2 and 8 [19].

Despite our ability to detect dramatic changes in gene expression at the *H. austinensis* fertilization boundary, these data pose major challenges in deciphering mRNAs that may play important roles in embryogenesis, as opposed to those associated mainly with housekeeping functions. To dissect these various roles, we focused on candidate zygotic transcripts selected unbiasedly by fold-expression difference, total count difference, and DeSeq2 algorithmic analysis [13], aided by KEGG. Concomitantly, we conducted a biased approach with known developmental regulators (e.g., homologues of nanos, bicoid, etc.) identified in other model organisms, mostly representing Ecdysozoa (e.g., *D. melanogaster*, *C. elegans*). Unexpectedly, these various approaches generated mostly non-overlapping sets of differentially expressed zygotic transcripts in *H. austinensis* zygotes (Stage 1).

Among the most highly expressed zygotic transcripts were cell maintenance genes, including those related to chromatin structure and cell cycle regulation, which resembled the profiles of other animals at the MZT (reviewed in [18]). Other zygotic transcripts, however, suggest a more direct role in embryogenesis, comprising a group of nucleic acid binding domains and transcription factors that are upregulated in Stage 1 *H. austinensis* zygotes (see Figure 4). For example, Pumilio homologues are thought to participate in RNA clearance [20], but are also associated with the zinc finger protein, nanos, to establish embryonic polarity in *Drosophila* [21]. A number of hypothetical proteins (e.g., *Helobdella* proteins A–F) appeared across our different selection criteria and are worth noting, but it is inherently difficult to make definitive assertions with this collective, baseline data. Likewise, our biased gene candidates showed that relatively few putative *H. austinensis* homologues were differentially expressed, or even represented, in our dataset, with some exceptions (i.e., Par and polarity homologues, nanos). Some inferences can be made with previous studies that identify *Hro-nos*, for example, as an early developmental regulator [22,23], as noted above, but other leech homologues (e.g., Rho1, Rho4) were not differentially expressed at the MZT, according to our data. This does not mean, however, that maternally deposited proteins corresponding to known developmental regulators are not acting at this developmental stage, since they would not be detected using our RNA-based analyses.

In conclusion, we demonstrate that major changes in the cellular RNA profile occur in *H. austinensis* upon zygote deposition, which likely prepares the embryo for subsequent developmental events, including the first asymmetric cleavage. These changes include a massive degradation of maternal RNAs coupled with the very early transcription of zygotic cell maintenance genes and putative developmental regulators. With our current data, we cannot determine whether embryonic asymmetry (e.g., cell polarity, first cleavage) is dependent on the initiation of zygotic transcription, but establishing the genetic composition of these first transcripts has allowed us to identify several gene targets for testing such hypotheses, as well as providing a baseline to explore other early developmental events in leeches.

## Figures and Tables

**Table 1:** Transcript counts with a 2-fold differential expression threshold.

Transcript Type	Unique Transcripts	Change in Total Transcript Count between Stage 0 → 1
Downregulated (degraded)	8295	−1,449,456
Upregulated (transcribed)	4979	+4,785,366
Stage 0—specific	3322	−93,259
Stage 1—specific	760	+1122

**Table 2:** BLAST results for selected early developmental genes.

Homologue	Helrodraft #	Query Coverage	E-Value	Identity	Stage 0 Count	Stage 1 Count
Par1	171,411	28%	0	79.23%	28	34
Par3	192,383	33%	5 × 10^−53^	31.02%	191	499
Par6	106,160	69%	3 × 10^−92^	59.68%	0	0
SKN-1	130,334	53.0%	2 × 10^−83^	45.45%	7	0
Bicoid	148,580	9.00%	1 × 10^−12^	46.55%	0	0
Hunchback	116,286	34%	3 × 10^−47^	54.49%	79	20
Caudal	112,342	81%	1 × 10^−165^	34.15%	128	250
Nanos	166,185	85%	1 × 10^−140^	48.11%	519	1114
Polarity protein	169,490	42%	4 × 10^−113^	56.15%	80	583
Rho1	167,950	100%	1 × 10^−126^	87.50%	2215	4302
Rho4	161,341	41.0%	2 × 10^−32^	40.0%	1786	2153
Wingless	163,913	66%	5 × 10^−53^	41.40%	0	0

## Data Availability

Data are contained within the article.

## Supplementary Materials

The following supporting information can be downloaded at: https://www.mdpi.com/article/10.3390/genes15030283/s1, Figure S1: Scatter plot of unique transcripts according to Log-10 counts in Stage 0 vs. Stage 1, with 5-fold and 10-fold thresholds. Each dot identifies a single, unique transcript color-coded according to the legend; Figure S2: (A) Twelve most downregulated maternal transcripts according to DeSeq2 statistical analysis. Dots identify independent transcriptomes from Stage 0 and Stage 1, respectively. (B) Twelve most downregulated maternal transcripts according to transcript count change. (C) Twelve most downregulated maternal transcripts according to fold-change; Table S1: Transcript counts at the 2-fold, 5-fold, and 10-fold thresholds.

## Abbreviations

BLAST	basic local alignment search tool
Hro-nos	*Helobdella robusta* nanos homologue
KEGG	Kyoto Encyclopedia of Genes and Genomes
MZT	maternal-to-zygotic transition
RNA	ribonucleic acid
