# Supplementary material for: Transcriptomic Profiling at the Maternal-to-Zygotic Transition in Leech, Helobdella austinensis
Source: Genes (Basel). 2024 Feb 24;15(3):283. doi: 10.3390/genes15030283 (PMC10970458; doi:10.3390/genes15030283)
Supplement: Supplementary file 1 [file genes-15-00283-s001.zip › genes-2877043-supplementary.pdf]

Supplementary Data

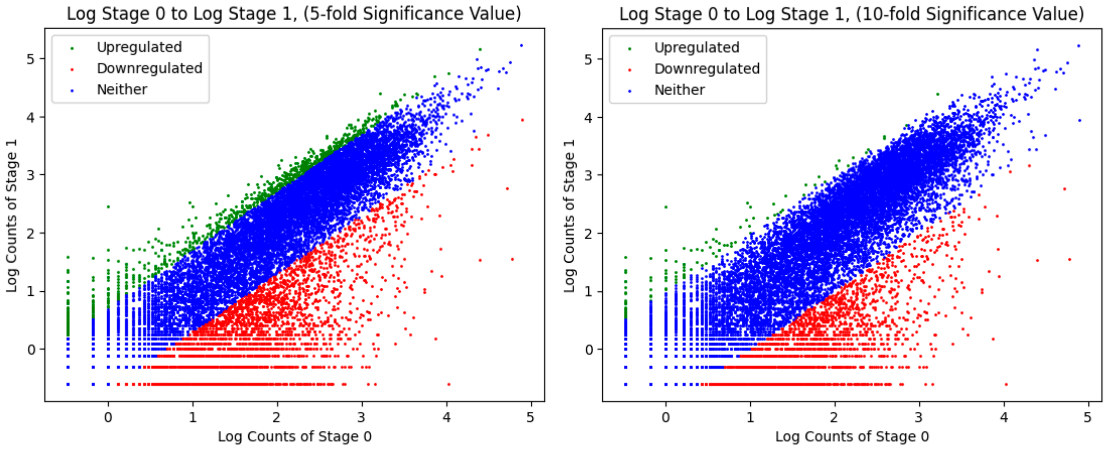

Figure S1: Scatter plot of unique transcripts according to Log-10 counts in Stage 0 vs Stage 1, with 5-fold and 10-fold thresholds. Each dot identifies a single, unique transcript color-coded according to legend.

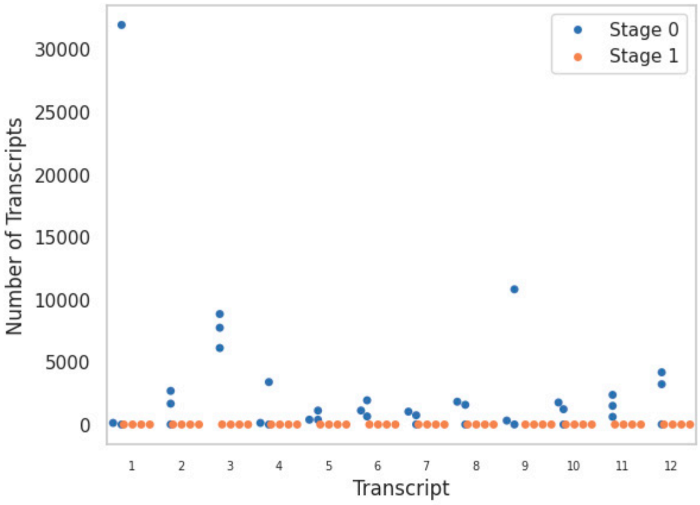

Lane	Annotation Name	Putative Function	E-Value	Query Percent	percent identity
1	HELRODRAFT_173735	ER lumen protein-retaining receptor-like isoform X1	1.00E-110	33%	76.89%
2	HELRODRAFT_168863	A disintegrin and metalloproteinase with thrombospondin motifs 3-like	1.00E-25	74%	25.09%
3	HELRODRAFT_95007	aquaporin	2.00E-103	57%	61.99%
4	HELRODRAFT_177712	MAM and LDL-receptor class A domain-containing protein 2-like isoform X3	2.00E-08	51%	30.32%
5	HELRODRAFT_132940	Multiple C2 and transmembrane domain-containing protein 1	1.00E-75	58%	46.10%
6	HELRODRAFT_100875	hemerythrin	4.00E-52	41%	70.83%
7	HELRODRAFT_189841	Otopetrin-3	8.00E-08	14%	32.16%
8	HELRODRAFT_181765	Hyp. *Helobdella* Protein A	0	71%	96.01%
9	HELRODRAFT_173706	carbonic anhydrase family protein	2.00E-21	55%	32.33%
10	HELRODRAFT_187798	neprilysin-like	2.00E-178	77%	38.80%
11	HELRODRAFT_188008	protein PLANT CADMIUM RESISTANCE 3-like	4.00E-26	25%	56.38%
12	HELRODRAFT_182419	Hyp. *Helobdella* Protein B	4.00E-153	56%	92.64%

Figure S2A. Twelve most downregulated maternal transcripts according to DeSeq2 statistical analysis. Dots identify independent transcriptomes from Stage 0 and Stage 1, respectively.

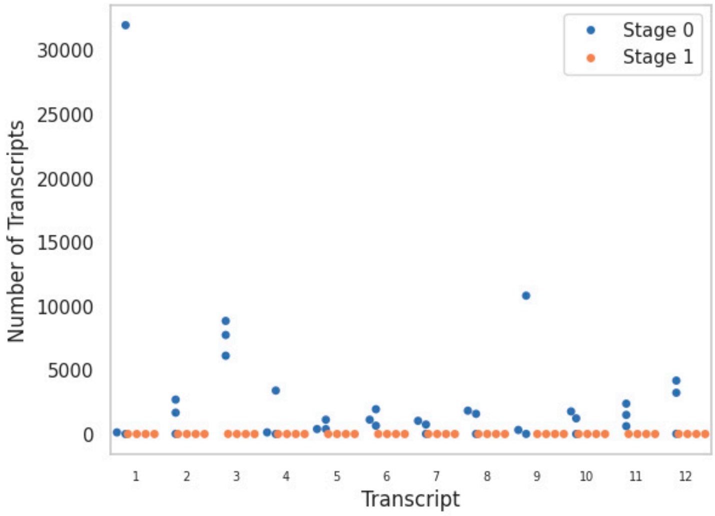

Lane	Annotation Name	Putative Function	E-Value	Query Percent	Percent Identity
1	HELRODRAFT_70460	collagen alpha-1(I) chain-like protein	2.00E-39	42%	61.70%
2	HELRODRAFT_186040	Hau-cytoplasmic intermediate filament 3, partial	0	37%	99.70%
3	HELRODRAFT_96235	actin, clone 403	0	28%	97.88%
4	HELRODRAFT_93044	putative 18s rrna sequence	1.00E-43	22%	60.78%
5	HELRODRAFT_191271	selenoprotein Pb-like	0.001	14%	28.33%
6	HELRODRAFT_175538	protein PRQFV-amide-like	2.00E-07	53%	38.35%
7	HELRODRAFT_105094	senescence-associated protein	7.00E-93	55%	72.67%
8	HELRODRAFT_185885	Checkpoint kinase 2	0	54%	98.85%
9	HELRODRAFT_186139	heat shock protein-70kDa	0	67%	89.71%
10	HELRODRAFT_178639	Hyp. *Helobdella* Protein A	6.00E-68	31%	95.37%
11	HELRODRAFT_156722	peptidyl-prolyl isomerase F (cyclophilin D), partial	4.00E-79	34%	78.24%
12	HELRODRAFT_185172	tubulin alpha-3 chain	0	51%	93.76%

Figure S2B. Twelve most downregulated maternal transcripts according to transcript count change.

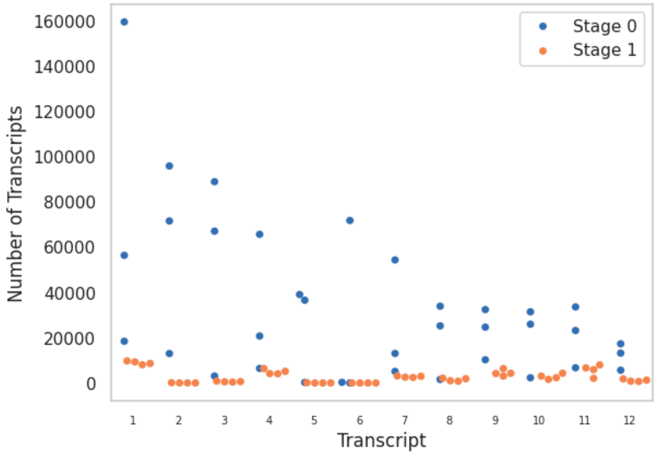

Lane	Annotation Name	Putative Function	E-Value	Query Percent	Percent Identity
1	HELRODRAFT_173735	ER lumen protein-retaining receptor-like isoform X1	1.00E-110	33%	76.89%
2	HELRODRAFT_168863	A disintegrin and metalloproteinase with thrombospondin motifs 3-like	1.00E-25	74%	25.09%
3	HELRODRAFT_95007	aquaporin	2.00E-103	57%	61.99%
4	HELRODRAFT_177712	MAM and LDL-receptor class A domain-containing protein 2-like isoform X3	2.00E-08	51%	30.32%
5	HELRODRAFT_132940	Multiple C2 and transmembrane domain-containing protein 1	1.00E-75	58%	46.10%
6	HELRODRAFT_100875	hemerythrin	4.00E-52	41%	70.83%
7	HELRODRAFT_189841	Otopetrin-3	8.00E-08	14%	32.16%
8	HELRODRAFT_181765	Hyp. *Helobdella* Protein A	0	71%	96.01%
9	HELRODRAFT_173706	carbonic anhydrase family protein	2.00E-21	55%	32.33%
10	HELRODRAFT_187798	neprilysin-like	2.00E-178	77%	38.80%
11	HELRODRAFT_188008	protein PLANT CADMIUM RESISTANCE 3-	4.00E-26	25%	56.38%
12	HELRODRAFT_182419	Hyp. *Helobdella* Protein B	4.00E-153	56%	92.64%

Figure S2C: Twelve most downregulated maternal transcripts according to fold-change.

Table S1. Transcript counts at 2-fold, 5-fold and 10-fold thresholds.

Threshold	Downregulated	Upregulated
2-fold	8,295	4,979
5-fold	2,956	836
10-fold	2,003	110
